# Identification and characterization of a novel *ELN* mutation in congenital heart disease with pulmonary artery stenosis

**DOI:** 10.1038/s41598-021-93736-1

**Published:** 2021-07-08

**Authors:** Cuilan Hou, Junmin Zheng, Wei liu, Lijian Xie, Xiaomin Sun, Yongwei Zhang, Meng Xu, Yun Li, Tingting Xiao

**Affiliations:** 1 Department of Cardiology, Shanghai Children’s Hospital, Shanghai Jiaotong University, No. 355 Luding Road, Shanghai, 200062 China; 2 NHC Key Laboratory of Medical Embryogenesis and Developmental Molecular Biology, Shanghai Key Laboratory of Embryo and Reproduction Engineering, Shanghai, 200062 China

**Keywords:** Biochemistry, Cell biology, Genetics, Pathogenesis

## Abstract

Congenital heart defects, one of the most common birth defects, affect approximately 1% of live birth globally and remain the leading cause of infant mortality in developed countries. Utilizing the pathogenicity score and inheritance mode from whole exome sequencing results, a heterozygous mutation (NM_001278939.1: c.1939G>T, p.Gly647Ter) in elastin (*ELN*) was identified among 6,440 variants in a female proband born with an atrial septal defect accompanied by pulmonary artery stenosis. Results of RT-PCR showed that the mutation (NM_001278939.1: c.1939G>T, p.Gly647Ter) did not affect the expression levels of *ELN* mRNA but increased protein level. The content of *ELN* truncate (functional component) was significantly lower in both the intracellular and extracellular compartments after mutation. These results indicate that the *ELN* mutation (NM_001278939.1: c.1939G>T, p.Gly647Ter) affected the protein truncate, which may be a functional component of *ELN* and play crucial roles for this pedigree. Here we report of an *ELN* heterozygous variant associated with congenital heart disease accompanied with pulmonary artery stenosis, which is less common. Based on our results, we speculate that this may be the main molecular mechanism underlying the mutation-led functional changes, and propose that the decrease of *ELN* protein level may cause this pedigree vascular abnormality, especially pulmonary artery stenosis, and reinforce the view that *ELN* insufficiency is the primary cause of these vascular lesions. This may be the main molecular mechanism underlying the mutation-led functional changes. Thus, systematic analysis not only enables us to better understand the etiology of this disease but also contributes to clinical and prenatal diagnosis.

## Introduction

Congenital heart defects (CHDs), one of the most common birth defects, affect approximately 1% of live birth globally and remain the leading cause of infant mortality in developed countries^1^. It has been reported that a strong genetic component, environmental causes, and familial forms play crucial roles in CHDs^2^. It has been estimated that de novo mutations in more than 400 genes likely contribute to ~ 10% of CHDs^3^, while chromosomal abnormalities are implicated in over 20% cases^4^. Heart failure is the most frequent complication of CHDs. With improved medical management and surgical outcomes, survival in cases of complex CHDs has improved significantly. However, many CHDs or complications with unknown etiology require urgent and targeted treatments.

Pulmonary artery stenosis is a narrowing (stenosis) that occurs in the pulmonary artery, which sends oxygen-poor blood into the lungs to be enriched with oxygen. There are four types of pulmonary artery stenosis: type I, type II, type III, and type IV^5^. CHDs, such as atrial septal defects, are sometimes accompanied with pulmonary artery stenosis. CHDs are mainly caused by a combination of genetic and environmental factors. Williams syndrome, is a rare hereditary disorder that affects many parts of the body, and it is most often due to deletions on chromosome 7q11.23. According to the ClinVar database (https://www.ncbi.nlm.nih.gov/clinvar) and the Human Gene Mutation Database (http://www.hgmd.cf.ac.uk), more than 100 pathogenic or presumed pathogenic variants of the Elastin (*ELN*) gene have been described^6^. The *ELN* gene sequence and its protein structure were first reported in 1985^7^ and amended in 1991^8^. Human *ELN* gene encodes for 786 amino acids and is composed of 34 coding exomes. It is well recognized that mutations in a functional domain or a protein translation modification site can alter protein function or protein–protein interaction^9^. *ELN* is the major structural protein of tissues such as the aorta and nuchal ligament, which must expand rapidly and recover completely. The *ELN* includes one conserved domain, Sporozoite_P67. The structure and function of *ELN* are not fully understood. The molecular defects in *ELN* have mainly been described in three conditions: supravalvular aortic stenosis^10^, autosomal-dominant cutis laxa^11^, and Williams syndrome^6^; it has rarely been reported *ELN* mutation in CHDs accompanied with pulmonary artery stenosis.

In this study, we report a female proband diagnosed of atrial septal defect accompanied with pulmonary artery stenosis. Whole exome sequencing (WES) was performed to identify possible disease-causing genes or variants. Paired-end reading was aligned with the GRCh37/hg19 human reference sequence. Through comprehensive Clinvar software and GATK analysis, BAM and VCF files were produced. An interesting heterozygous mutation of *ELN* (NM_001278939.1: c.1939G>T, p.Gly647Ter), identified in this female infant, is discussed along with the possible mechanism of gene mutation. Our results indicate that *ELN* mutation may be involved in CHDs accompanied with pulmonary artery stenosis and should be screened in prospective clinical practice.

## Materials and methods

### Clinical information and Ethics

A female infant was born with an atrial septal defect (ASD). There was no fever, cyanosis, cough, vomiting, or diarrhea, and no special treatment was given at that time. Subsequently, regular follow-up and reexamination were conducted in the outpatient department of our hospital. No signs or symptoms were identified during physical examinations. Ethics documentation of this case was waived with the approval of the Shanghai Children’s Hospital Institutional Review Board. The parents of the patient provided written informed consent for publication. All of the following studies were performed in accordance with the guidelines and regulations of the Ethics Committee of Experimental Research of Shanghai Children’s Hospital, Shanghai Jiaotong University.

### WES

DNA library construction and WES assays were carried out in accordance with the manufacturer's instructions. Briefly, the genomic data of the patient and her parents were collected using a whole-blood genomic DNA extraction kit (Tiangen, China), and 1 µg DNA was used for the WES assay. The precise experimental procedures and experimental instruments are described in detail in our previous study^12^.

### Sanger sequencing and mutation analysis

*ELN* mutation was confirmed via Sanger sequencing. Primers were designed using Primer 5 software to cover the known mutation sequence. The sequence of the forward primer was 5′-CCACTAGGAACTCCAGTTCTTC-3′, and that of the reverse primer was 5′-GGTCAGGCTGGTCTGGAACC-3′. PCR products were resolved and purified using the QIAquick kit (Qiagen, Germantown, MD USA). Sanger sequencing was carried out at Suzhou Hong Xun Biotechnology Co., Ltd.

The filtered and analyzed data are described in detail in our previous study ^12^. Briefly, data were filtered by self-developed software, and compared with the human genome database (GRCh 37/hg 19) using BWA-0.710 software. The single base mutation and insertion deletion mutation were identified, and compared with the 1,000 Genomes Project, Exome Aggregation Consortium databases, Exome Variant Server, gnomAD, Clinvar (http://www.ncbi.nlm.nih.gov/clinvar), OMIM, HGMD, and 370 samples of whole exome sequencing in our hospital. Variant filtration was performed after all variants were obtained, annotated, and assessed from the exome sequencing process. The mutation sites were evaluated with the COBALT homology alignment of amino acid sequences online tool among different species. The predicted effect of variants on protein function and conservation across species was assessed using SIFT, Polyphen-2, GERP (genomic evolutionary rate profiling), Mutation Taste, and combined annotation-dependent depletion. After identifying candidate genes, the frequency of the variant in the Exome Aggregation Consortium (ExAC; http://exac.broadinstitute.org) was reviewed. Rare variants for validation were polymerase chain reaction amplified. Illumina Variant Studio was used to filter the variants as per their frequency and presence or absence in the affected family members versus the healthy individuals.

### Plasmid construction and transfection

A DNA fragment containing full-length *ELN* cDNA was obtained by PCR amplification. The primer sequences are listed in Table 1 (ELN-F, R). Plasmid pEGFP-C1 was purchased from Bioeagle Biotech Company, Ltd, Wuhan, China. Restriction sites and full-length *ELN* (besides of introne) were inserted into the pEGFP-C1 plasmid to construct the recombinant vector pEGFP-ELN-wt. Using site-directed mutation kit (Fast site-directed mutagenesis kit, Tiangen, Beijing), the *ELN* mutation site (NM_001278939.1: c.1939G>T, p.Gly647Ter) was introduced in the above recombination carrier to construct the recombination carrier pEGFP-ELN-mut. The primer sequences used in these experiments are listed in Table 1 (ELN-F1, R1). The above two recombination plasmids were confirmed by sequencing.

**Table 1:** The primers used in construction of the plasmid, point mutation, and qPCR.

ELN-F	AACTCTAGAGAATTC GCCACCATGGCGGGTCTGAC
ELN-R	CTTCCATGGCTCGAG TCATTTTCTCTTCCGGCCAC
ELN-F1	ACCCAGGGTACCTTGAGCCCTGGCTGCCGCTAAAGCAGCCAAATATGGAGCA
ELN-R1	GCTTTAGCGGCAGCCAGGGCTCAAGGTACCCTGGGTGATGAGGGGGTGCTGGGGA
ELN-QPCR-F	GTCGGAGGGCTTGGAGTTC
ELN-QPCR-R	GACAATCCGAAGCCAGGTCT

Human embryonic kidney 293T cells were purchased from the Cell Bank of the Chinese Academy of Sciences and maintained in a humidified incubator maintained at 37 °C and 5% CO_2_ atmosphere. 293T cells were transiently transformed with pEGFP-ELN-wt/mut plasmid using Lipofectamine 2000 (Invitrogen, Carlsbad, CA, USA). Transfected 293T cells were cultured on a confocal dish, and the expression of pEGFP-ELN-wt/mut was examined using an inverted fluorescence microscope (Zeiss, Germany) with excitation at 488 nm and emission at 507 nm. Total RNA and proteins were extracted and verified by real-time-PCR (qPCR) and western blotting.

### Real-time PCR (RT-PCR)

RT-PCR was used to detect the expression of ELN mRNA. Briefly, 293T cells were transiently transformed with pEGFP-ELN-wt/mut plasmids, and the RNA of the cells was extracted according to the manufacturer’s protocol (Takara, Japan). The primer sequences are listed in Table 1(ELN-qPCR-F, R).

### Translation inhibition

A translation inhibitor cycloheximide (CHX) was used to detect the *ELN* translation^13^. Briefly, the constructed wild-type and mutant-type eukaryotic recombinant expression vectors were transiently transfected into human embryonic kidney 293 T cells then treated with cycloheximide (500 μM) for 0, 4, 8, and 24 h. Cell proteins were extracted and analyzed by western blotting.

### Western blotting

Western blotting method was described in detail in our previous work^14^. Proteins were collected and quantified using the BCA reagent (Thermo Fisher Scientific, Waltham, MA, USA). The proteins were resolved on a sodium dodecyl sulfate 10% polyacrylamide gel, transferred onto a polyvinylidene fluoride membrane (Millipore, Bedford, MA, USA), and incubated with primary antibodies (1:1000 dilution) against *ELN* (ABclonal, Wuhan, China) and GFP (Cell Signaling Technology, Danvers, MA, USA) at 4 °C overnight. Blots were incubated with secondary antibodies for another hour at room temperature. After washing, the blots were visualized using a chemiluminescent substrate, and then analyzed by image J software.

### Statistical analysis

Results are expressed as the mean ± SEM. Statistical analysis was performed using SPSS software, version 21.0 (SPSS, Inc., Chicago, IL, USA). Comparisons among groups were performed using one-way ANOVA. Paired data were evaluated by two-tailed Student’s *t*-test. Statistical significance was considered when *P* < 0.05.

## Results

### General clinical information and mutation characteristics

**Figure 1:**
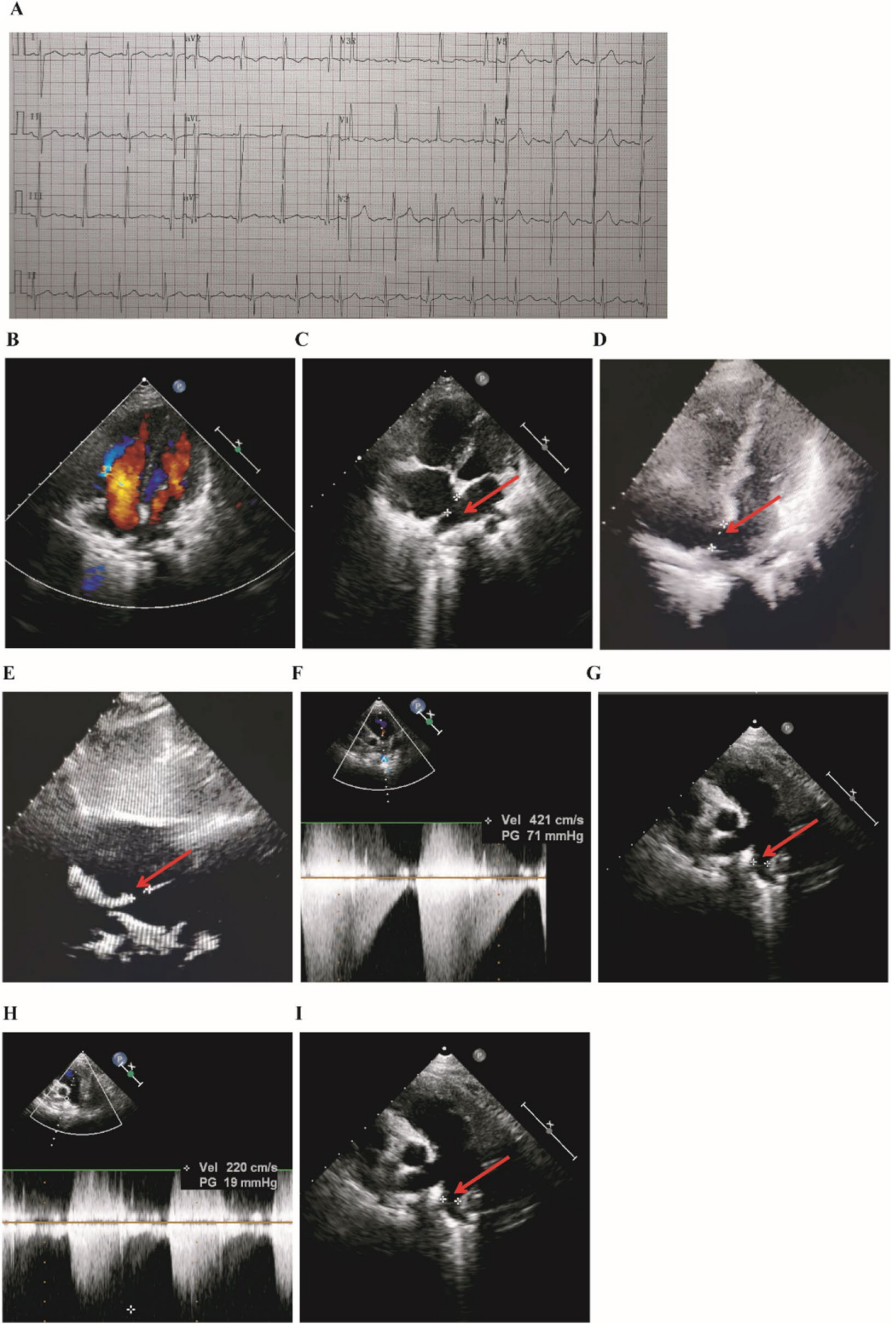
12-lead ECG and ultrasonic cardiogram in the patient. (**A**) 12-lead ECG in resting time showing sinus arrhythmia; slight hypertrophy (RV5/SV1 = 1.51/0.00 mV) evident in the right ventricular. (**B**–**I**) Ultrasonic cardiogram of the patient. The red arrow indicates the site of an atrial septal defect in the left and right pulmonary arteries.

A 12-lead electrocardiogram (ECG) during the resting time showed sinus arrhythmia and minor hypertrophy in the right ventricle (Fig. 1A). Biochemical metabolism, myocardial enzyme, and cardiac computed tomography angiography were also performed and showed normal results. Further ultrasound cardiogram showed that the atrial septum was continuously interrupted, with a size of approximately 1.20 cm × 1.56 cm (Fig. 1B–E). The internal diameter of the right pulmonary artery opening was 1.14 cm, and the blood flow was 2.2 m/s. The internal diameter of the left pulmonary artery opening was 0.8 cm, the flow rate was 4.21 m/s, and the pressure difference was 71 mmHg (Fig. 1F–I). The infant showed symptoms of atrial septal defect and pulmonary artery stenosis.

**Figure 2:**
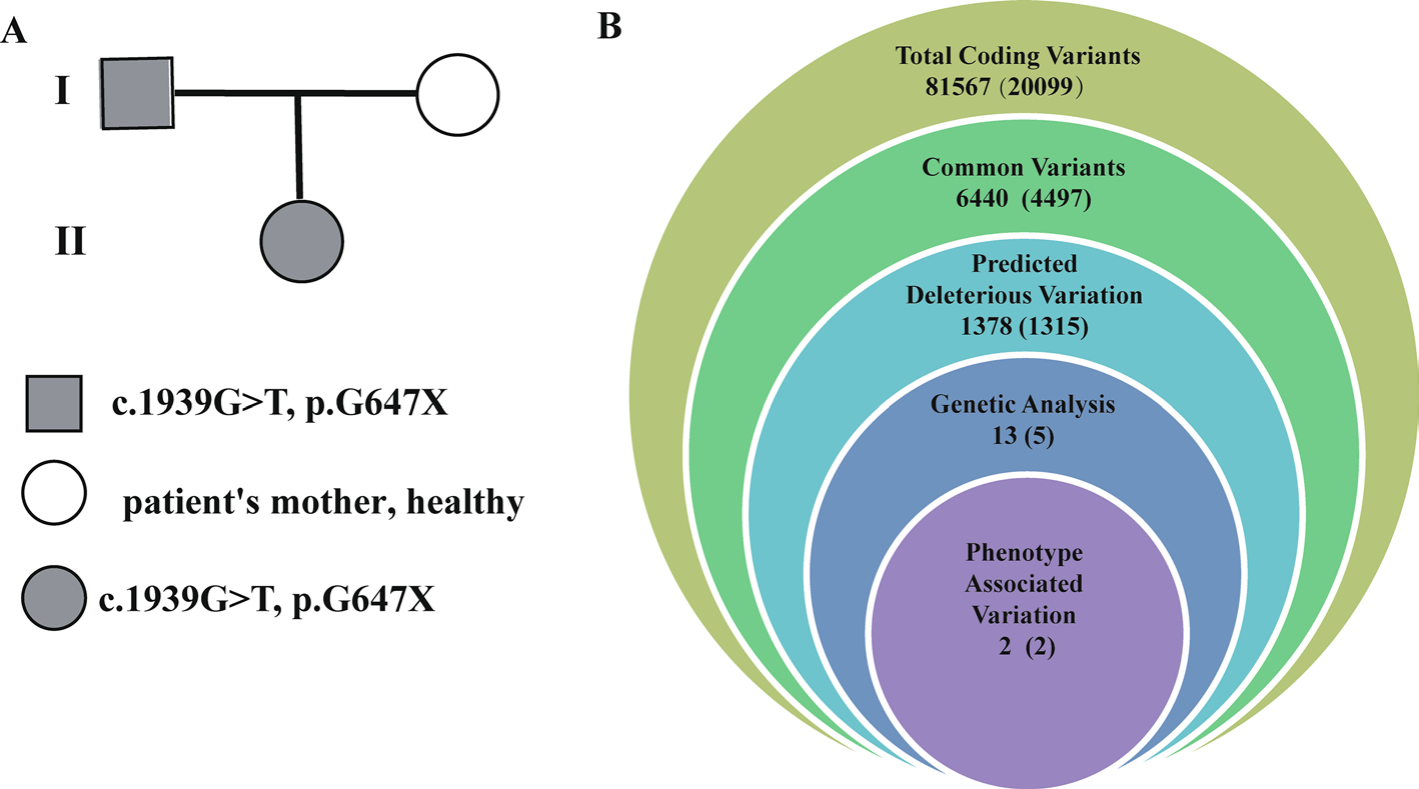
Family pedigree and the filtering process for WES data. (**A**) Family pedigree consists of one proband. I-1 represents the proband’s father; I-2 represents the proband’s mother; II-1 represents the proband. (**B**) The filtering process for WES data, containing 81,567 total coding variants. Filtering results: 1315 deleterious variations, two variants selected from genetic analysis, final gene associated with this phenotype variation.

As shown in Fig. 2A,B, the outermost layer, 81,567 mutation sites, the total number of mutations detected from 20,999 genes, which was annotated and filtered by Ingenuity Variant Analysis (MAF < 0.05) according to the standards of the Exome Aggregation Consortium, 1,000 Genomes Project, Exome Sequencing Project, or gnomAD. In the second layer, 6,440 mutations were found in 4,497 genes screened for population frequency. In the third layer, 1,378 mutations were found in 1,315 genes at sites predicted to be pathogenic. In the fourth layer, 13 mutations were found in 5 genes, which were analyzed based on co-segregation of families. Fifth layer, 2 mutations occur in 2 genes (*ELN* and *CHRNG*), phenotypic related genetic variations. Lastly, we find that *ELN* was the most consistent with the phenotype, the *ELN* mutation was identified and selected after rigorous analysis linked to the infant phenotype (Fig. 2B). These rare phenotype-related variants were classified following the guidelines of the American College of Medical Genetics and Genomics/Association for Molecular Pathology.

**Figure 3:**
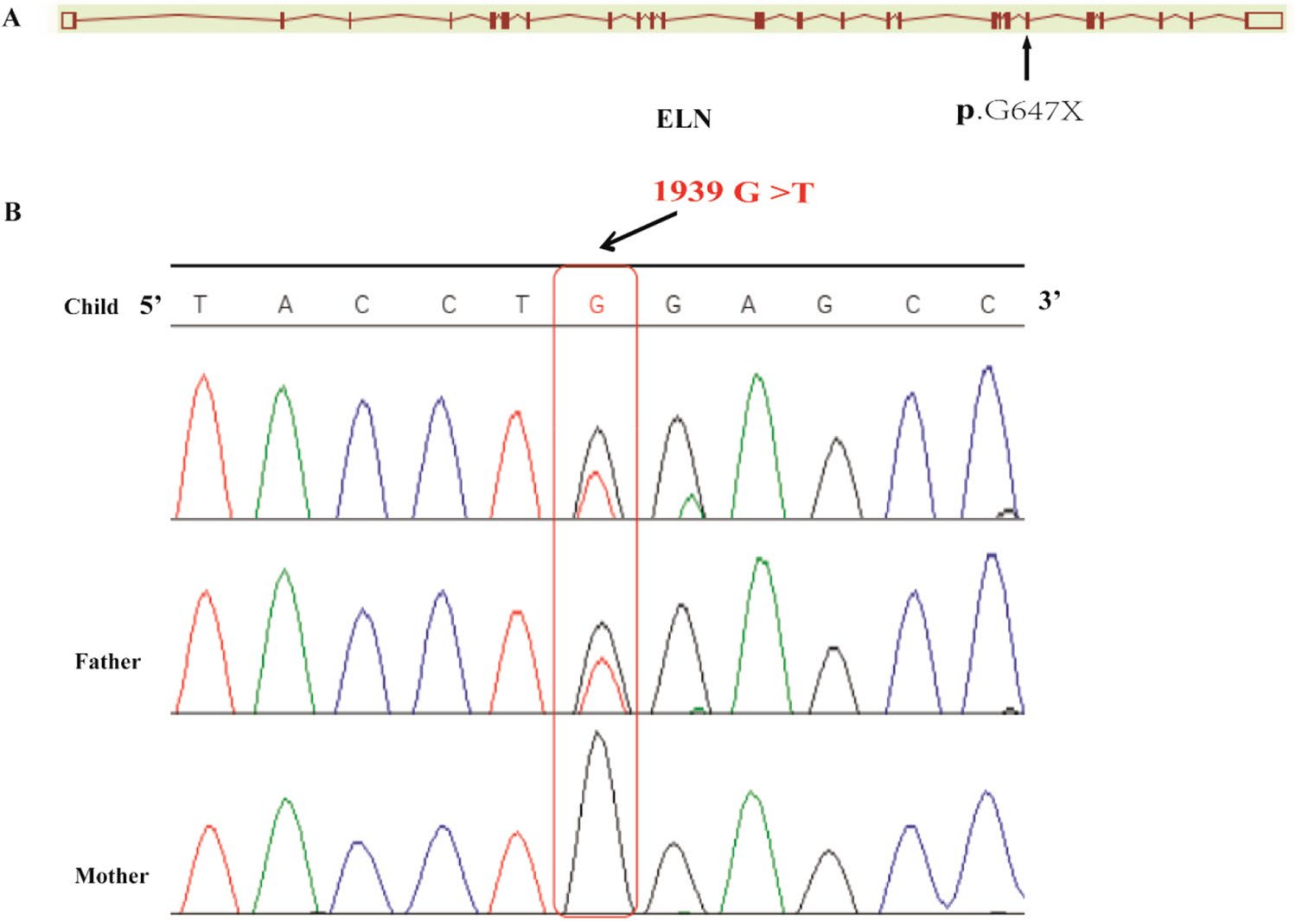
The *ELN* mutation site and its conservation. (**A**) Human *ELN* gene maps to chromosome 7q11.23 and contains 34 exomes. The base pair mutation site is c.1939G>T, which is located on the exome 27 of *ELN*. (**B**) The proband and her father were confirmed to be heterozygous carriers of 1939G>T hybridization, and her mother was homozygous negative for this mutation, as shown by Sanger sequencing.

A heterozygous mutation of the *ELN* gene (NM_001278939.1: c.1939G>T, p.Gly647Ter) was associated with a CHD with pulmonary artery stenosis pedigree through WES. The mutation (NM_001278939.1: c.1939G>T, p.Gly647Ter) is located in exon 27 of *ELN*. The infant and her father were confirmed to be heterozygous carriers of 1939G>T (NM_001278939.1: c.1939G>T, p.Gly647Ter), and her mother was homozygous negative for the mutation as shown through Sanger sequencing (Fig. 3A,B).

### *ELN* mutation analysis

**Figure 4:**
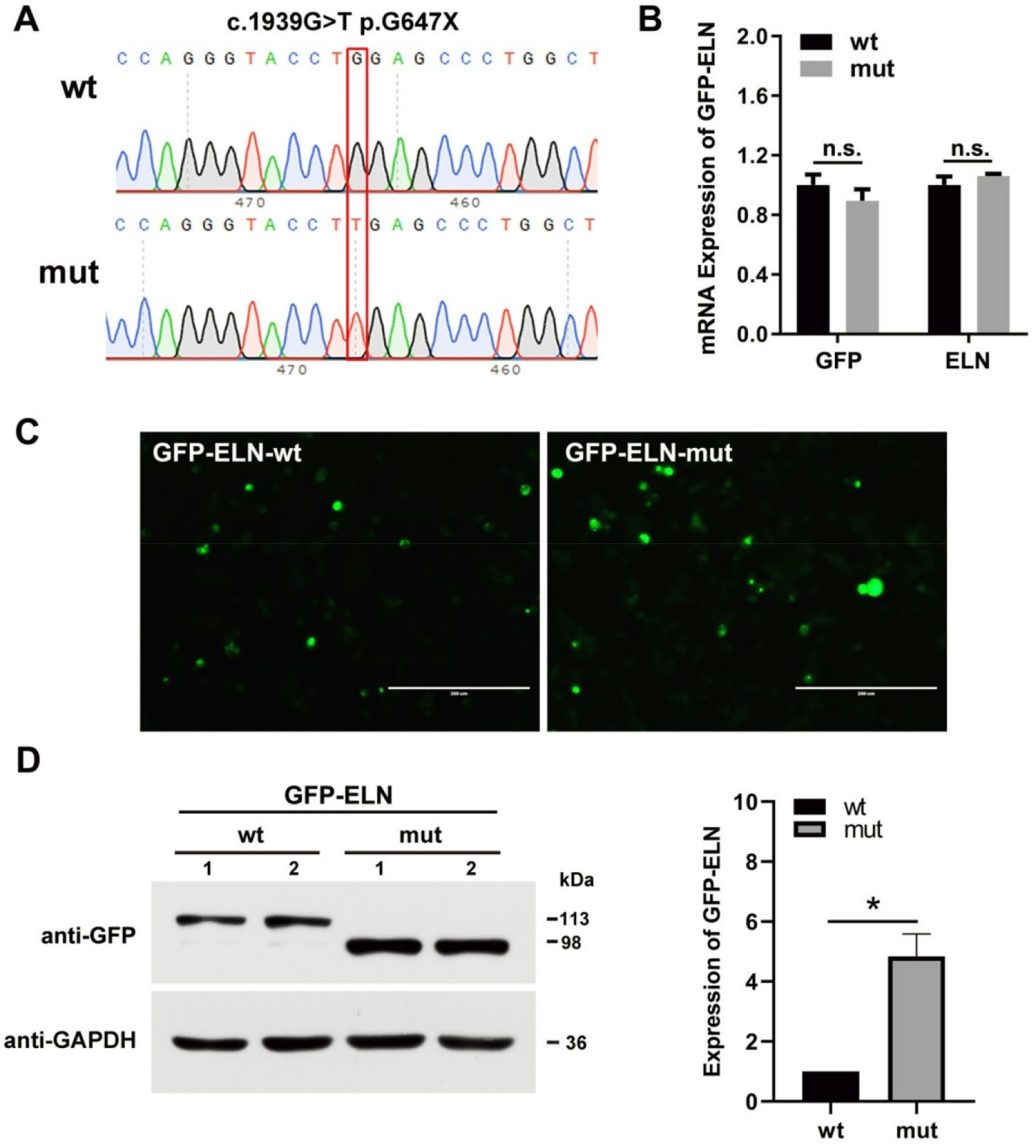
The mutation site (NM_001278939.1: c.1939G>T, p.Gly647Ter) affects *ELN* expression. (**A**) The pEGFP-ELN-wt/mut sites as showed through Sanger sequencing. (**B**) *ELN* mRNA levels before and after mutation. (**C**) Micrographs of fluorescence microscope images of *ELN* in 293 T. Scale bar, 200 µm. *ELN* was stained with green, the nucleus with blue (N = 3). (**D**) Immunoblot of GFP protein levels and quantification of *ELN* relative protein levels in 293 T. GAPDH served as loading control. Values are means ± SEM. *P < 0.05, **P < 0.01 was considered significant (N = 6).

RT-PCR was utilized to detect expression of *ELN* mRNA. 200 µL of whole-blood sample was used to extract RNA according to protocol (PrimeScript™ RT Master Mix, takara). Primers were designed before and after the mutation to explore whether the mutation altered its expression. Before the mutation forward primer: 5′-AGCTAAAGCAGCAGCAAAGT-3′ and reverse primer: 5′-CTGCAGCAGCTCCATACTTG-3′. After the mutation forward primer: 5′-CTTGGAGTTCCAGGTGTTGG-3′ and reverse primer: 5′-TGGGAAAATGGGAGACAATC-3′. We find that *ELN* mRNA levels remained unchanged before and after mutation. The pEGFP-ELN-wt/mut sites are shown in Fig. 4A. Results of RT-PCR showed that *ELN* mRNA expression levels did not change after mutation, which suggests that the mutation did not affect *ELN* expression at the mRNA level (Fig. 4B). The results of the relative fluorescence intensity of GFP and the western blotting suggested that the mutation (NM_001278939.1: c.1939G>T, p.Gly647Ter) increased *ELN* protein levels. The pEGFP-ELN-wt group *ELN* staining indicated a molecular weight of approximately 113 kDa, whereas the pEGFP-ELN-mut group *ELN* staining showed a molecular weight of approximately 98 kDa (Fig. 4C,D). The RT-PCR and western blotting indicated that the mutation (NM_001278939.1: c.1939G>T, p.Gly647Ter) can lead to increasing intracellular *ELN* protein expression.

**Figure 5:**
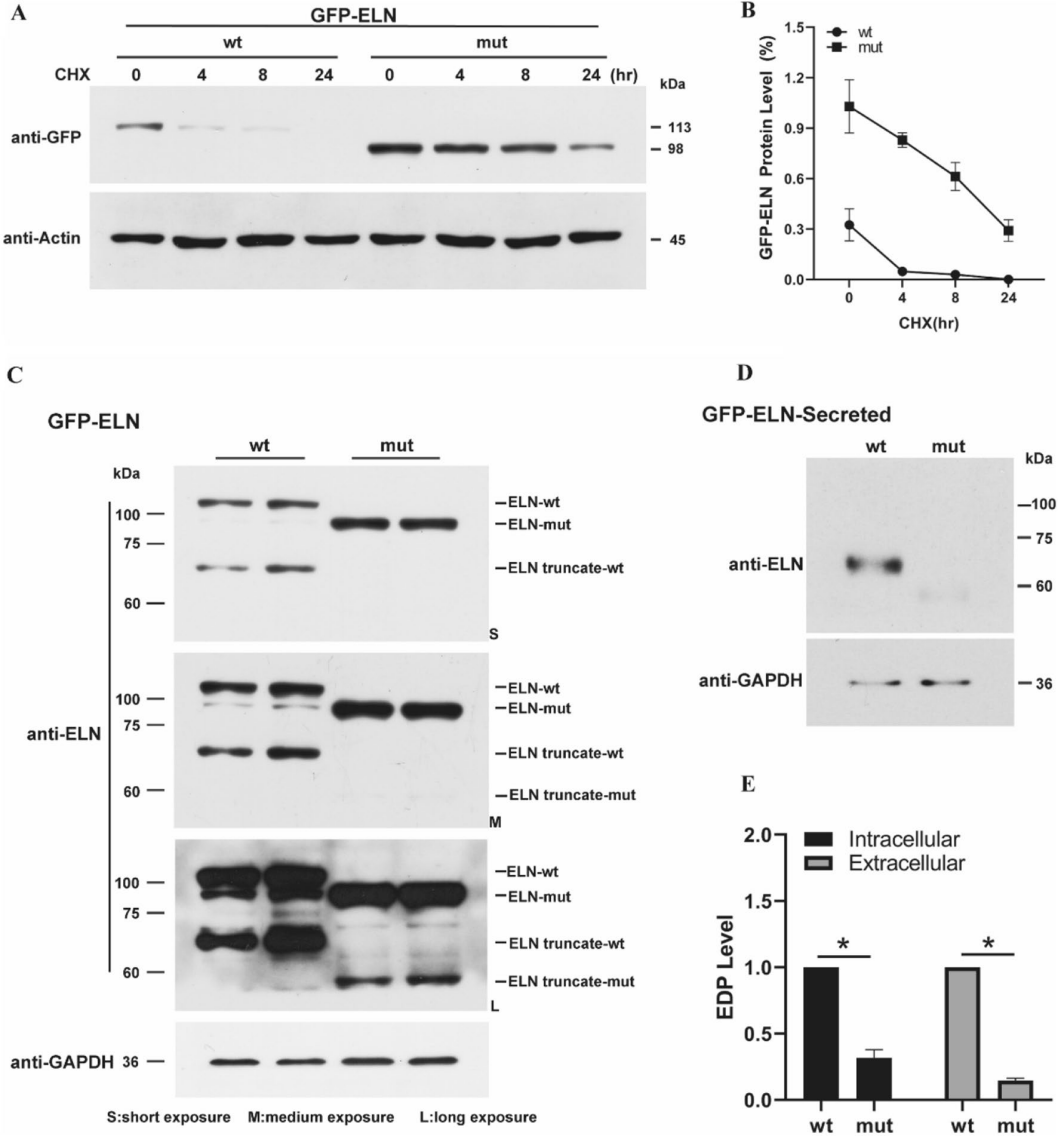
Effects of CHX on *ELN* expression and its relative secreted expression. (**A**) Effects of CHX on *ELN* expression in transfected cells. Immunoblot of GFP protein levels between the pEGFP-ELN-wt and pEGFP-ELN-mut group after incubation with CHX (500 μM) for 0, 4, 8, and 24 h. (**B**) Quantification of relative *ELN* protein levels in transfected cells. (**C**) The relative quantity of *ELN* and its truncated body under different exposure times. (**D**) Immunoblot of secreted *ELN* protein levels. (**E**) Quantification of *ELN* shear body relative protein levels. Values are means ± SEM. *P < 0.05, **P < 0.01 was considered significant (N = 5).

After 4 h of treatment with CHX, the *ELN* expression level of the pEGFP-ELN-wt group was significantly reduced and was difficult to detect after 24 h. However, the degradation of the pEGFP-ELN-mut group was considerably slower, and the protein bands were visible even after 24 h (Fig. 5A,B). Different exposure times showed the relative quantity of *ELN* and *ELN* truncate. The band showing larger molecular weight indicates full-length expressed proteins, such as pEGFP-ELN-wt at 113 kDa and pEGFP-ELN-mut at 98 kDa. With the increase in exposure time, protein truncates (68 kDa and 53 kDa bands) were noted in the two groups, and these two truncated body proteins were expected to be the *ELN* truncates. The relative amount of *ELN* truncates (68 kDa and 53 kDa bands) decreased after introduction of the mutation (Fig. 5C). These results suggest that the mutation may affect the processing of *ELN* protein, which occurs before its exocytosis. Millipore Amicon ultra-4 10K was used to concentrate approximately 6 mL cell culture super plasma to 200 μL, after which the relative expression of *ELN* protein was again detected by western blotting. In these extracellular samples, no full-length *ELN* bands, namely 113 kDa and 98 kDa bands, were detected; only the *ELN* truncate, namely 68 kDa and 53 kDa bands, were found (Fig. 5D). The exocytosis of *ELN* in the pEGFP-ELN-wt group was significantly higher than that in the pEGFP-ELN-mut group (Fig. 5E).

Taken together, our results shown that the mutation (NM_001278939.1: c.1939G>T, p.Gly647Ter) did not affect the mRNA expression and that the expression level of *ELN* was significantly higher in mutated variants than that of the wild-type group. However, the content of *ELN* truncates (functional component) was significantly lower in both the intracellular and extracellular compartments than in the wild-type group.

## Discussion

In this study, clinical phenotype and genotype of a mutated CHD with pulmonary artery stenosis pedigree were collected and analyzed to investigate a potential disease-causing variant. Three interesting findings are as follows: (1) The heterozygous mutation of *ELN* (NM_001278939.1: c.1939G>T, p.Gly647Ter) is found in this pedigree; (2) The mutation did not alter *ELN* gene mRNA levels, while increase its protein levels; (3) The content of *ELN* truncate (functional component) was significantly lower both in intracellular and extracellular than in the wild-type group. The *ELN* (NM_001278939.1: c.1939G>T, p.Gly647Ter) mutation could affect its functional active component expression, and may play a crucial role in this case.

It is well established that extensive heterogeneity of CHDs is due to a combination of genetic and environmental factors that serve as phenotype modulators. *TBX5*, *NKX2.5*, *GATA4*, *ZIC*, *MYH6*, *NOTCH1*, *CRELD1*, and *FOG2* have been reported to be associated with CHDs^15,16^. Within the known non-syndromic CHD cases, the incidence of causative gene mutations is less than 7%, which hinders the understanding of the disease mechanism and the development of treatment strategies.

To explore the possibility of the role of functional effects caused by the *ELN* mutation (NM_001278939.1: c.1939G>T, p.Gly647Ter) on disease occurrence, we constructed both wild type and mutant plasmids of *ELN* and analyzed the *ELN* mRNA and protein levels. We found that the mutation site had no significant impact on its transcriptional level, while it significantly increased the protein levels (Fig. 4). The *ELN* heterozygous variant (NM_001278939.1: c.1939G>T, p.Gly647Ter), is located in the exon 27 and plays a vital role in modulating elastin. Humans are extremely sensitive to reduced *ELN* expression, and develop profound arterial thickening, which in turn markedly increases the risk of obstructive vascular disease^17,18^. Moreover, the aorta, septum, and pulmonary artery contains high levels of elastin, indicating that the *ELN* heterozygous variant may be one of the probable reasons for this pedigree.

A paradoxical increase in arterial elasticity along with abnormalities in elastic fibers was observed in patients with Williams–Beuren syndrome^19^. To investigate why a deletion mutation in the ELN gene causes an inherited obstructive arterial disease, such as supravalvular aortic stenosis, Li et al. generated a transgenic mouse (Eln^+/−^). They found that, during the arterial development, *ELN* hemizygosity in mice and humans induces a compensatory increase in the number of rings of elastic lamellae and smooth muscle. Humans are sensitive to reduced *ELN* expression, development to deep arterial thickening and significantly increased risk of obstructive vascular disease^20^. Micale Lucia et al. then used minigene and cycloheximide experiments to show that some selected frameshift mutant alleles (c.1161delC, c.838_839insG, c.1195delG) are the substrates of nonsense-mediated mRNA decay (NMD), which confirmed that the functional haploinsufficiency of the *ELN* gene is the main pathological mechanism of supravalvular aortic stenosis. Their results indicate the importance of screening for the *ELN* gene in patients with vascular abnormalities, especially SVAS and pulmonary artery stenosis, and reinforce the view that haploinsufficiency at *ELN* is the primary cause of these vascular lesions^21^.

In the present study, the mutation site increased *ELN* protein level (Fig. 4D), which is not normally seen in this pedigree (pulmonary artery stenosis). This proband is diagnosed with ASD accompanied with left and right pulmonary artery stenosis. It is well recognized that *ELN* mutation may act via impacting protein dosage and function, and may cause diseases such as supravalvular aortic stenosis^10^, autosomal-dominant cutis laxa^11^, and Williams syndrome^6^. In non-syndromic supravalvular aortic stenosis with a nonsense mutation, mRNA degradation of mutant alleles leads to a large numbers of premature termination codon mutations in *ELN*, resulting in insufficient *ELN* levels^22^. While reviewing the literature, we found that increased protease activity in the elastin-rich tissues leads to elastin degradation^23^. The peptides produced due to the degradation (elastokines, elastin-derived peptides, and elastin-related peptides) have been shown to be biologically active^17^. Their activities can be either physiologically beneficial or part of a pathological process. Accordingly, we increased the exposure time and found truncated body proteins (68 kDa and 53 kDa) in both pEGFP-ELN-wt and pEGFP-ELN-mut groups, and the relative amount of truncated body proteins (68 kDa and 53 kDa) decreased after the mutation effect (Fig. 5C). These results indicate that the mutation may affect the processing of *ELN* protein, which occurs before exocytosis of *ELN* protein.

Next, we detected the protein content secreted in the cell culture medium. The concentration of the cell culture medium indicated that there was no full-length *ELN* protein in the two groups, and the expression of truncated *ELN* decreased in the mutant group (Fig. 5D,E). In the arteries of patients with Williams-Beuren syndrome, increased proliferation of arterial smooth muscle cells in a quiescent contractile state is due to decreased deposition of elastin^22^. Therefore, beyond elasticity, elastin can also act as an autocrine factor in smooth muscle cells. A reduction in *ELN* expression was also observed in cutaneous fibroblasts and aortic smooth muscle cells in affected non-syndromic supravalvular aortic stenosis patients, thus, supporting the role of *ELN* haploidy as a pathogenesis of vascular lesions^24^. Overall, the content of truncated *ELN* (functional component) processed after translation was significantly lower both in intracellular and extracellular compartments after mutation. Our data are consistent with previous findings^21^. We propose that the decrease of *ELN* protein level may cause this pedigree vascular abnormality, especially pulmonary artery stenosis, and reinforce the view that *ELN* insufficiency is the primary cause of these vascular lesions. This may be the main molecular mechanism underlying the mutation-led functional changes.

In conclusion, genetic diagnosis of CHDs before the onset of symptoms is essential. The *ELN* heterozygous mutation (NM_001278939.1: c.1939G>T, p.Gly647Ter) was associated with the CHD accompanied with a pulmonary artery stenosis family. *ELN* truncate (functional component) was significantly lower both in intracellular and extracellular regions, and this may be the main molecular mechanism responsible for the mutation leading to the disease phenotype. Systematic analysis not only allows us to gain a better understanding of this disease etiology, but also contributes to clinical and prenatal diagnosis.
